# Specificity of Interaction between *Clostridium perfringens* Enterotoxin and Claudin-Family Tight Junction Proteins

**DOI:** 10.3390/toxins2071595

**Published:** 2010-06-24

**Authors:** Leslie A. Mitchell, Michael Koval

**Affiliations:** 1 Division of Pulmonary, Allergy and Critical Care Medicine, Department of Medicine, 205 Whitehead Bldg, 615 Michael St. Emory University School of Medicine, Atlanta, GA 30322, USA; Email: leslie.mitchell@emory.edu; 2 Department of Cell Biology, Emory University School of Medicine, Atlanta, GA 30322, USA

**Keywords:** *Clostridium perfringens*, claudin, tight junction, intestinal epithelium, cancer therapeutics, acute lung injury

## Abstract

*Clostridium perfringens* enterotoxin (CPE), a major cause of food poisoning, forms physical pores in the plasma membrane of intestinal epithelial cells. The ability of CPE to recognize the epithelium is due to the *C-*terminal binding domain, which binds to a specific motif on the second extracellular loop of tight junction proteins known as claudins. The interaction between claudins and CPE plays a key role in mediating CPE toxicity by facilitating pore formation and by promoting tight junction disassembly. Recently, the ability of CPE to distinguish between specific claudins has been used to develop tools for studying roles for claudins in epithelial barrier function. Moreover, the high affinity of CPE to selected claudins makes CPE a useful platform for targeted drug delivery to tumors expressing these claudins.

## 1. Introduction

*Clostridium perfringens* is a rod-shaped, Gram-positive, anaerobic bacterium, which is responsible for a significant fraction of food borne disease [1,2]. There are five subclasses of *C. perfringens*, which are classified based on the relative expression of alpha, beta, epsilon, and iota toxin. Of these subclasses, a fraction of subclass A clinical isolates produce a 35 kDa polypeptide known as *C. perfringens* enterotoxin (CPE) [3]. Although CPE is not required for intestinal pathogenicity, subclasses of *C. perfringens* that express CPE use this protein to exacerbate the pathogen's effects on intestinal epithelia. The increased pathogenicity is due to the ability of CPE to specifically interact with a subclass of tight junction proteins, known as claudins [4,5,6]. Although there are over two dozen different claudins, CPE has been shown to interact with claudin-3, -4, -5, -6, -7, -8, -9 and -14 to varying degrees, and does not recognize other claudins. As a result, CPE has emerged as a tool being used to elucidate roles for claudins in epithelial barrier function. In addition, CPE-based agents are being tested for use as a targeted therapeutic, most notably as anti-tumor drugs.

## 2. CPE Receptors Are Claudin Family Tight Junction proteins

Fibroblasts lack the ability to bind CPE and are resistant to CPE-mediated cell death. Thus, fibroblasts were used to screen a cDNA library for constructs which confer sensitivity to CPE toxicity. Two high affinity CPE transmembrane protein receptors (CPE-R and RVP-1) were discovered using this approach [7,8]. It was only a few years later that Morita *et al.* [9] revealed CPE-R and RVP-1 to be the tight junction proteins claudin-4 and claudin-3, respectively. This finding added CPE to the growing list of proteins produced by pathogens that use different classes of host junction proteins as receptors [10,11,12]. 

**Figure 1:**
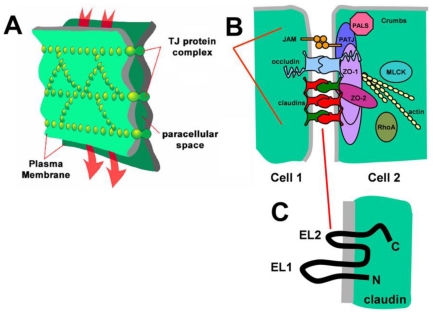
Protein components oftight junctions between polarized epithelial cells. (**A**) Based on freeze fracture electron microscopy, tight junctions appear as a series of beaded strands in the plasma membrane at cell-cell contact sites, modified from [13]; (**B**) Tight junctions consist of several proteins, including transmembrane proteins linked to the actin cytoskeleton by scaffold proteins. Of these transmembrane proteins, claudins are the primary structural determinants of paracellular permeability; (**C**) Claudins span the bilayer four times, with *N-* and *C-* termini oriented towards the cytosol and have two extracellular loop (EL) domains.

Claudins are 20–27 kDa transmembrane proteins that span the membrane bilayer four times (Figure 1). The *N*- and *C-* termini are oriented towards the cytoplasm and there are two extracellular loop domains, both of which mediate interactions with other claudins [14,15,16]. Claudins are a major constituent of tight junctions, which are cell-cell contact sites between polarized epithelial cells that serve to regulate the movement of ions and molecules between cells. In addition to claudins, tight junctions also contain other proteins, including transmembrane proteins such as occludin and cytoplasmic scaffold proteins, mainly zonula occludens (ZO)-1 and -2, which tether claudins to the cytoskeleton and are required to maintain paracellular permeability [17,18,19]. 

Although several different types of protein are needed to regulate tight junctions, it is primarily the profile of claudin expression which enables tight junctions to have unique permeability by forming what are effectively paracellular channels [20]. Paracellular ion selectivity is established by structural motifs in the two claudin extracellular loop domains, primarily charged amino acids in the first extracellular loop (EL1) [15,21,22,23,24]. By contrast, the smaller second extracellular loop (EL2) contains ~25 amino acids and helps narrow the paracellular gap and plays a role in regulating heteromeric claudin-claudin interactions [25]. In addition, the claudin EL2 domain is the site where CPE binds to specific claudins (see below).

## 3.  Mechanism of Action for CPE Toxicity

After ingestion of contaminated food, *C. perfringens* passes from the stomach to the small intestine where it multiplies and sporulates. During sporulation, CPE is expressed and accumulates within the bacterium until it is discharged when the sporulating cells lyse. Upon release into the intestinal lumen, CPE binds to intestinal epithelial cells and initiates a cascade of events leading to cell death [7,8,26].

**Figure 2:**
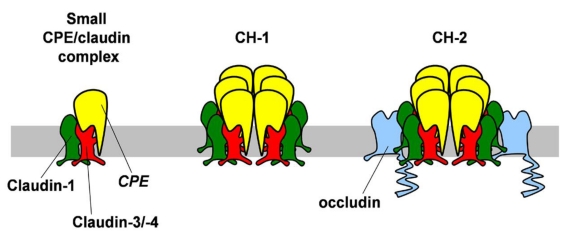
Sequential intermediates in CPE:epithelial cell membrane interactions. CPE initially binds directly to claudins at the plasma membrane, most typically claudin-3 or claudin-4 on intestinal epithelium. The initial small CPE/claudin complexes may also include other claudins, e.g., claudin-1, via an indirect interaction. Six small complexes oligomerize to form a hexameric complex (CH-1) that forms pores which compromise plasma membrane integrity. CH-1 complexes eventually incorporate occludin and disrupt epithelial tight junctions resulting in a breakdown of barrier function.

There are three major biochemically isolatable complexes that represent the sequence of interaction of CPE with cell tight junction proteins, a small complex containing CPE and claudins which subsequently oligomerizes and then incorporates occludin [27]. CPE initially binds to claudins to form a small 90 kD complex (Figure 2), which itself is insufficient for cytotoxicity [26]. Interestingly, when formed by human CaCo2 colon epithelial cells, the small CPE complex contains claudin-1 in addition to claudin-3 and/or claudin-4, despite the fact that CPE does not directly bind to claudin-1 [28,29]. Thus, the presence of claudin-1 in the complex is more likely to be due to an interaction with claudin-3 and/or claudin-4, rather than a direct interaction with CPE. In addition to claudin-1, it is likely that other claudins expressed by intestinal epithelial cells can also associate with the small CPE/claudin complex. In fact, claudin-5 has been isolated from CPE-affinity columns using normal rat cholangiocytes as a starting material, which could be enhanced by an indirect interaction via other claudins [30]. Based on apparent size, the small CPE/claudin complex contains one molecule of CPE (35 kD) and two claudin molecules (2 × 22 kD = 44 kD). The stoichiometry of the small CPE-claudin complex is intriguing in light of current models where claudins stably oligomerize as hexamers [31] and suggests that CPE either disrupts claudin hexamers or it interacts with claudin monomers or dimers at a step prior to complete claudin oligomerization. In the latter case, this would imply that claudin oligomerization is completed at the plasma membrane, perhaps following assembly into tight junctions. However, the sequence of claudin assembly remains to be determined [4]. Nonetheless, claudin-3 mutants which are unable to heterotypically interact have an increased ability to bind to CPE, underscoring that CPE interacts with claudins prior to incorporation to tight junction strands [32].

Small CPE/claudin complexes then combine to form a large (~450 kD) complex (CH-1) required for cytotoxicity [33]. These hexameric pores increase plasma membrane ion permeability [34] allowing calcium influx that induces cell death by apoptosis or oncosis [35,36].

A ~25 amino acid hydrophobic hairpin domain of CPE is required to form a beta-barrel pore in the plasma membrane [37]. The hairpin domain is similar to domains in other beta-barrel pore forming bacterial toxins that oligomerize in complexes ranging from pentamers to octomers [38]. However, while these other toxins require cholesterol for membrane binding and pore formation, CPE does not [39]. In fact, CPE binding induces claudins to partition away from cholesterol enriched membrane microdomains, thus contributing to the disruption of epithelial barrier function. Whether claudins contribute to CPE pore structure is not known, however, since claudins oligomerize as part of their role in tight junction formation, they may be a structural element of the CPE pore. It is interesting to note that a *C-*terminal claudin-4 mutant which lacks the PDZ-binding motif, a domain required for binding to ZO-1 and ZO-2 and incorporation into tight junction strands, still supports small complex formation and CPE-mediated toxicity. Thus, tight junction scaffold proteins are not required for CPE-claudin interactions or pore formation [33].

CH-1 complexes have also been shown to mature into larger CH-2 complexes containing occludin [40]. The interaction of CPE with occludin requires claudins, although a low affinity occludin binding site of CPE has not been ruled out. Binding of CPE to claudins and occludin is associated with internalization of these tight junction proteins, a process that compromises epithelial barrier function and therefore contributes to the mechanism of action for CPE toxicity [40]. Consistent with this model, internalization and subsequent degradation of tight junction proteins is frequently associated with impaired barrier function [41,42,43]. It remains to be determined whether CH-2 induces claudin and occludin internalization or forms after endocytosis to promote tight junction protein degradation.

## 4. Structural Basis and Specificity of CPE-Claudin Interactions

Further screening of claudins using transfected fibroblasts and/or biochemical approaches was used to determine that full length CPE or CPE-derived protein fragments can bind to claudin-6, -7, -8, -9 or -14 [28,32,44]. Moreover, this analysis confirmed that several claudins were unable to interact with CPE, which defined a subset of claudin-family proteins with the capacity to act as CPE receptors. While CPE can interact with several different claudins *in vitro* and in transfected cells, the pathobiology of *C. Perfringens* depends upon the claudins expressed by intestinal epithelium and accessibility to toxins. In addition to claudin-3 and claudin-4, other claudins significantly expressed by intestinal epithelium, which can also bind CPE, include claudin-7 and claudin-8 [45,46], however whether these or other claudins are involved in disease caused by *C. Perfringens* is unknown at present.

**Figure 3:**
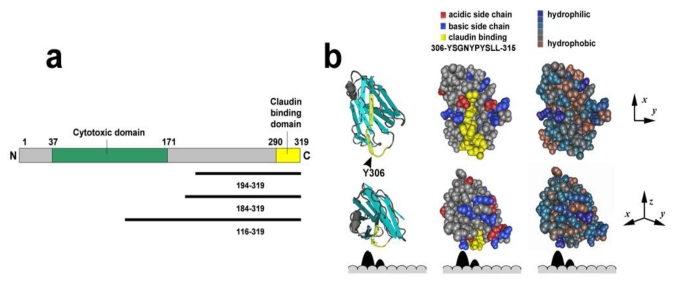
Claudin binding motif of CPE. (**a**) Linear diagram showing the domain structure of CPE, emphasizing the cytotoxic (green) and claudin binding (yellow) domains. Key truncation mutants are shown below; (**b**) Structural models of the *C-*terminal CPE claudin binding domain (CPE_194–319_) [47] were produced using the Molecular Modeling Database (MMDB) [48,49]. Shown from left to right are a ribbon diagram, and space filling models indicating amino acid charge or hydrophobicity. The top row shows CPE_194–319 _with the claudin binding motif facing the reader, in the bottom row the protein is rotated 90°. Shown in yellow is the claudin binding motif of CPE, which is enriched for hydrophobic amino acids. The arrowhead denotes the position of Tyr^306^. Below each row is a representation of the claudin extracellular loops, EL1 and EL2, where EL2 interacts with CPE.

Structure-function analysis of CPE reveals that it is comprised of an *N-*terminal cytotoxic domain and a *C-*terminal binding domain. Because a truncation mutant lacking the *N-*terminal half of the protein (CPE_171–319_) did not exhibit cytotoxic effects when applied to sensitive cells, Hanna *et al.* [50] concluded that the *N-*terminal region of CPE was required for toxicity. However, removal of the first 37 amino acids of the protein increases the cytotoxicity of the molecule [51,52]. 

While CPE_171–319_ is not toxic to cells, this truncated protein retains the capacity to bind to claudins [50]. Further truncation demonstrated that the last 30 amino acids (CPE_290–319_) are sufficient to recognize some claudins [53]. In addition, a synthetic peptide corresponding to these 30 amino acids was shown to have competitive binding activity equal to the native toxin [54], even though it lacks the cytotoxic domain needed for cytolysis, suggesting that all of the CPE receptor-binding activity is mediated by these residues [53]. However, other motifs might modulate the specificity of claudin binding. For instance CPE_116–319_ binds to cells expressing claudin-5, albeit at low affinity, while CPE_194–319_ does not [32]. 

The crystal structure of CPE_194–319_ shows that it is a nine-strand sandwich with similarities to the receptor binding domains of other pore-forming toxins (Figure 3) [47]. Surface electrostatic potential modeling shows that these residues form an acidic cleft surrounding a hydrophobic valley [44]. Consistent with a role for hydrophobic residues in stabilizing the interaction of CPE binding with claudins, mutating Tyr^306^ to Lys, a positively charged residue, completely abolishes claudin binding, while mutations to another aromatic residue, Phe, had no effect [55]. While it is possible that the Lys substitution destabilizes CPE and causes it to assume an alternative conformation, it does not seem likely given that Tyr^306^ is located on an intervening surface loop between two beta strands (Figure 3, arrowhead). Mutating Tyr^306^, Tyr^310^, or Tyr^312^ to Ala also impairs CPE claudin interactions [44], while mutation of Tyr^306^ in combination with one of the other two key Tyr residues completely eliminates claudin binding. This mutational analysis underscores the significance of bulky hydrophobic amino acids for high affinity CPE-claudin binding [56]. 

Fujita *et al.* [28] first demonstrated that CPE binds to claudins via EL2 using fibroblasts transfected with claudin-1/-3 chimeras, using sensitivity to CPE toxicity as an assay for binding. A comparable analysis also showed that the EL2 domain of claudin-7 was required for CPE-mediated toxicity; further refinement of the CPE binding site using point mutants defined a key role for Asn^149^ of claudin-4 in binding to CPE [44,57]. 

The EL2 binding sites of claudins with high affinity for CPE (claudin-4, claudin-3, claudin-7) have a calculated pI in the range of 6.4–9.7 and are enriched for amino acids which can interact with acidic residues in the cleft region of the claudin binding site of CPE [44]. In further define roles for basic residues in CPE-claudin interactions, a double point mutant in the EL2 domain of human claudin-5 was developed, where Asp^149^ was replaced with Asn and Tyr^158^ was replaced with Arg. These amino acid substitutions increased the calculated pI of the EL2 domain from 4.2 to 9.7 and significantly increased the affinity of binding to full length CPE as compared to wild type human claudin-5 [44]. Thus, electrostatic interactions between CPE and claudins promote the specificity of binding.

**Figure 4:**
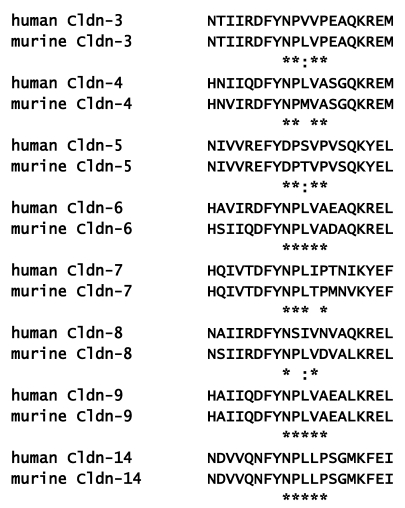
Extracellular loop domains of claudins recognized by CPE. The NP(L/V)(L/V)(P/A) binding motif within EL2 is highlighted below. Note that human claudin-8 shows significant sequence divergence from murine claudin-8 and is unlikely to interact with CPE. *—conserved between human and murine claudin, **:**—comparable amino acid substitution.

Using an array of peptides corresponding to the EL2 claudin domains, Winkler *et al*. [32] quantified binding of a GST-CPE_116–319_ fusion protein. This analysis was used to identify a key motif, NP(V/L)(V/L)(P/A), in the turn region of EL2 as a required sequence needed for claudins to interact with CPE (Figure 4) [32]. The EL2 CPE binding motif is usually conserved when comparing human and murine claudins (Figure 4). An important exception to this rule is human claudin-8 which does not interact with CPE since it lacks the NPLVD motif present in murine claudin-8 [32,44]. 

By and large, there was good agreement between the ability of GST-CPE_116–319_ to bind the EL2 peptide fragment and binding of full length CPE to full length claudins expressed by cells. However, the murine claudin-4 EL2 peptide was unable to bind to GST-CPE_116–319_, despite the ability of the CPE peptide to recognize claudin-4 in transfected cells [32,57]. It is noteworthy that the murine claudin-4 EL2 domain contains Met in the third position of the CPE binding motif; this Met residue may destabilize the claudin-4 EL2 peptide and prevent it from attaining a fully native conformation *in vitro*, despite being tolerated in full length claudin-4 expressed by cells. Also, CPE_116–319_ binds to an EL2 peptide from murine claudin-3 with greater affinity than a murine claudin-7 peptide [32], yet full length CPE equivalently recognizes HEK293 cells transfected with either claudin-3 or claudin-7, based on cell killing assays [44]. Differences in the affinity of CPE for isolated EL2 domains *vs.* full length claudins *in situ*, indicates the plasticity of EL2 conformation and underscores the need for high resolution claudin structure determination to fully understand CPE-claudin interactions.

## 5. CPE as a Tool to Study Epithelial Tight Junctions

The binding of CPE to specific claudins affects the structure of epithelial tight junctions independent of its cytotoxic effects [27,29,58]. This property is shared by *C-*terminal CPE fragments which retain the capacity to bind claudins. For instance, Sonoda *et al.* [29] showed that addition of the CPE_184–319_ fragment to the basolateral surface of MDCK I cells selectively removed endogenous claudin 4 from tight junctions while largely maintaining claudin-1 localization. Additionally, CPE_184–319_ treatment led to fragmented tight junctions and impaired barrier function, indicating a central role for claudin-4 in maintaining MDCK barrier function [29]. 

The recent observation that claudin-4 is specifically upregulated in response to acute lung injury led to the use of CPE as a tool to identify specific roles for claudin-4 in the injury response [58]. In this study, Wray, *et al.* [58] used a CPE_290–319_ peptide administered *in vivo* to mice prior to experimentally induced lung injury of varying severity. CPE_290–319_ administered *in vivo* significantly decreased claudin-4 content of the lung. Functionally, CPE_290–319_ dramatically increased bulk alveolar protein permeability (leak) in response to severe lung injury, underscoring a role for upregulated claudin-4 in protecting the lung from mechanical injury to tight junctions. By contrast, alveolar protein permeability was low and largely unaffected by CPE_290–319_ in both the unstressed lung and lungs subjected to moderate injury. However, CPE_290–319_ treatment increased the amount of lung edema in response to moderate ventilator induced lung injury and, importantly, diminished the fluid clearance capacity of unstressed lungs. Thus, CPE_290–319_ provided the first *in vivo* evidence in support of a role for claudins in regulating the fine control of fluid balance in the lung [59]. Since lungs express several claudins which can interact with CPE, including claudin-3 and -7 [42,60,61], it is possible that changes in the mouse lung physiology induced by CPE_290–319_ are due to effects on other claudins. However, the relatively mild effect of CPE_290–319_ on unstressed lungs where claudin-4 is not upregulated argues against this possibility [58].

Beyond identifying roles for claudin-4 in lung physiology, CPE-derived reagents could potentially be used to more generally study other aspects of epithelial tight junctions. In order to achieve this, CPE variants would need to be designed which preferentially recognize different claudins beyond the subset of claudins which already bind CPE. This requires understanding of the molecular basis for CPE-claudin interactions to a level of depth allowing informed design of new CPE variants.

## 6. Targeted Cancer Therapeutics Using CPE

Histological screens of human tumors have revealed changes in claudin expression associated with tumor phenotype [62,63]. Altered claudin expression may lead to abnormal barrier function which, in turn, can increase paracellular permeability to ultimately facilitate tumor cell mobility and promote nutrient supply to tumor cells [64,65,66,67]. Consistent with a reduction in the tight junction barrier, claudin expression by tumor cells is frequently down-regulated [62,63,68,69,70,71,72,73,74,75,76,77]. 

However, there are also several examples where claudin expression is upregulated in many tumors including breast, ovarian, and prostate cancers [62,63,78,79,80]. While this result may seem surprising given the role of claudins in tight junction integrity, recent work suggests that claudins are also involved in survival and invasion of cancer cells, functions that could be independent of their role as tight junction proteins [81,82,83]. Of particular interest, claudin-3 and claudin-4 seem to be generally upregulated, particularly in aggressively metatastic tumors [84,85]. 

Increased expression of claudin-3 or claudin-4 provides a fortuitous target in these tumors, since expression enables CPE-based therapeutic agents to be developed as potential anti-tumor agents [62,78,86]. Current preclinical therapies include use of both cytotoxic CPE alone and *C-*terminal fragments of CPE as a targeting molecule [78,86,87,88,89,90,91]. 

*In vitro*, CPE treatment of human cancer cell lines and, importantly, breast tumor tissue has been shown to result in cytolysis and cell death in a dose-dependent manner [78,86,87]. Using murine xenograft models, the *in vivo* efficacy of CPE therapy has also been tested. Intra-tumoral injection of CPE into pancreatic, breast, and brain cancer tumors significantly impeded tumor growth or caused tumor regression by inducing necrosis [78,86,88]. Furthermore, intraperitoneal injection of CPE significantly inhibited growth of explanted human ovarian cancer in mice without severe gastrointestinal side-effects or weight loss [87]. 

In addition to utilizing CPE to directly induce cytotoxicity, the non-cytolytic CPE_184–319_ fragment may be effective in the targeted delivery of chemotherapeutic agents. For instance, a fusion protein of CPE_290–319 _and TNF was used to induce cytotoxicity in ovarian cancer cells expressing both claudins-3 and -4 while remaining non-toxic to cells lacking these proteins [90]. Recently, Ebihara *et al.* fused CPE_194–319 _to the protein synthesis inhibitory factor (PSIF) domain of *Pseudomonas* exotoxin (C-CPE-PSIF) and found that this fusion protein was toxic to human breast cancer cells that express claudins-3 and -4 [89]. Furthermore, this fusion protein had no effect on L cells expressing exogenous claudin-1, claudin-2 or claudin-5 or SK-HEP-1 hepatocytes lacking claudin-4 but expressing other claudins, such as claudin-1 [92]. 

Two features of C-CPE-PSIF are critical for its mechanism of action. First, by binding to claudins, CPE fusion proteins are internalized by endocytosis [89]. Second, internalized C-CPE-PSIF is proteolyzed to release a free PSIF fragment which subsequently crosses the membrane bilayer into the cytosol where it kills cells by inhibiting protein synthesis [93]. Importantly, the efficacy of C-CPE-PSIF was recently demonstrated *in vivo*, using several murine tumor metastasis models [94]. In this study, C-CPE-PSIF decreased the growth rate of subcutaneous tumors derived from cells expressing claudin-4 by 50% and, in the case of 4TI cell derived tumors, the number of metastases found in the lungs was almost completely repressed.

Although the ability of CPE or CPE-based fusion proteins to bind to isolated circulating metastatic cells seems straightforward, the mechanisms by which externally applied CPE can successfully target solid tumors is less clear. The ability of CPE to cause a breakdown in tight junction barriers is expected to contribute to penetration into tumors [29,92,95]. Alternatively, the anti-tumor effect of CPE may be limited to a superficial layer of cells in cases where growth is inhibited, but tumors do not decrease in mass [86]. It may well be that an adjunct therapeutic, such as the recently discovered iRGD peptide [96], will be needed to optimize the efficacy of CPE-based anti-cancer drugs.

The inherent toxicity of full length CPE represents a significant side effect with the potential to reduce therapeutic efficacy. The route of administration has a significant effect on toxicity. For instance, intraperitoneal injection is significantly more toxic than intragastral administration or direct injection into tumors [86,78,97]. For sub-lethal doses of CPE, both intraperitoneal or intragastral administration induces a transient spike in cytokine production (mainly IL-6 and IFN-γ) which peaks at 8 h post treatment [97]. There are also data that CPE acts as a direct mitogen for macrophages and T cells *in vitro* [97,98] and could have superantigen activity [99]. However, other studies demonstrated that D-Galactose induced liver injury did not sensitize mice to CPE toxicity [97] and CPE does not act as a mitogen for peripheral leukocytes *in vitro* [100], both of which are hallmarks of superantigenicity. These conflicting data are likely to reflect differences in CPE preparations, if contaminants contribute to the immune response. From a practical standpoint, some concerns related to CPE-mediated toxicity are likely to be ameliorated by replacing the cytotoxic domain of CPE with a domain that has a more specific mode of action, such as PSIF, particularly if this prevents a systemic immune response. 

Taken together, these studies support the use of CPE-based pharmaceuticals as a strategy to specifically target tumors with a suitable profile of claudin expression. In general, claudins have great potential as targets for cancer therapy, but studies of this family are incomplete. While significant progress has been made in understanding the structural basis of CPE-claudin interactions, further analysis could enable other claudin specific interacting molecules to be designed to improve the specificity and efficacy of CPE-based therapeutic approaches.
